# Spectral Computed Tomography Angiography in Visceral Artery Aneurysms: Technical Principles and Clinical Applications

**DOI:** 10.3390/tomography12020022

**Published:** 2026-02-10

**Authors:** Laura Maria Cacioppa, Michaela Cellina, Giacomo Agliata, Francesco Mariotti, Nicolo’ Rossini, Tommaso Valeri, Giangabriele Francavilla, Alessandro Felicioli, Alessandra Bruno, Marzia Rosati, Roberto Candelari, Chiara Floridi

**Affiliations:** 1 Department of Clinical, Special and Dental Sciences, University Politecnica delle Marche, 60126 Ancona, Italytommi.val@libero.it (T.V.);; 2 Division of Interventional Radiology, Department of Radiological Sciences, University Hospital “Azienda Ospedaliera Universitaria delle Marche”, 60126 Ancona, Italy; 3 Radiology Department, ASST Fatebenefratelli Sacco, 20121 Milan, Italy; 4 Division of Radiology, Department of Radiological Sciences, University Hospital “Azienda Ospedaliera Universitaria delle Marche”, 60126 Ancona, Italy

**Keywords:** Computed Tomography Angiography, Dual-Energy Computed Tomography, spectral computed tomography, visceral artery aneurysm, endovascular aneurysm repair, endoleak, image reconstructions

## Abstract

Background: Visceral artery aneurysms (VAAs) are rare but potentially life-threatening vascular lesions often clinically silent until rupture. The widespread use of advanced imaging has increased incidental detection, highlighting the need for accurate, noninvasive diagnostic strategies. Dual-Energy Computed Tomography Angiography (DECTA) offers potential advantages over conventional CT across diagnostic and post-treatment settings; however, its role in VAAs remains incompletely defined. This narrative review summarizes current evidence on DECTA applications in VAAs, focusing on diagnosis, emergency evaluation, and post-treatment follow-up. Methods: A non-systematic literature search of PubMed and Embase focusing on English-language articles up to June 2025 was performed. The search included peer-reviewed original research articles, systematic reviews, and meta-analyses addressing dual-energy CT and spectral CT in vascular and aneurysmal imaging. Case reports without technical data and non-English articles were excluded. Results: In the diagnostic phase, DECTA enhances tissue differentiation through virtual monoenergetic images, iodine maps, and material decomposition reconstructions. In the post-treatment setting, DECTA supports assessment after endovascular procedures, including coil embolization or stent graft placement. In VAAs, these techniques may improve aneurysm delineation, reduce metal artifacts after endovascular treatment, enable accurate detection of endoleaks or residual perfusion, and support volumetric follow-up. Virtual Non-Contrast images may reduce radiation exposure without compromising diagnostic confidence. Conclusions: DECTA represents a versatile imaging modality with potential benefits across the diagnostic, emergency, and post-treatment phases of VAA management. Although many applications are extrapolated from aortic and peripheral vascular disease, emerging evidence supports its growing clinical relevance. Further dedicated studies are needed to define its role in VAA-specific decision-making and follow-up.

## Simple Summary

Visceral artery aneurysms (VAAs) are rare but potentially life-threatening arterial lesions, often detected incidentally. This review summarizes current evidence on Dual-Energy Computed Tomography Angiography (DECTA) for VAAs. DECTA can improve aneurysm visualization, support post-treatment assessment after endovascular procedures, reduce radiation exposure, and detect endoleaks or residual perfusions. Our review highlights the growing potential of DECTA in clinical practice while emphasizing the need for further studies to define its role in diagnosis, follow-up, and treatment decision-making for VAAs.

## 1. Introduction

Visceral Artery Aneurysms (VAAs) are uncommon vascular lesions, characterized by focal dilatations of the splanchnic arterial branches [1]. Although often asymptomatic and incidentally detected, VAAs carry a substantial risk of rupture, associated with high morbidity and mortality [1,2]. Early identification of VAAs is therefore essential to prevent potentially fatal outcomes, as well as their accurate post-treatment follow-up [1,2,3].

In recent years, Dual-Energy Computed Tomography Angiography (DECTA) has emerged as a valuable imaging modality, offering advanced diagnostic capabilities that may help to address several challenges associated with the evaluation and management of VAAs. The ability of Dual-Energy Computed Tomography (DECT) to acquire data simultaneously at two different energy levels (typically low and high kV) enables material composition analysis [4,5]. Post-processing techniques further enhance the utility of DECT enabling the generation of monoenergetic reconstructions, iodine maps, Virtual Non-Contrast (VNC) images [6,7,8]. In vascular settings, DECT can enhance the visualization of vessel wall and vascular structures and differentiate between plaque and thrombus components [9]. The use of monoenergetic imaging and metal artifact reduction algorithms significantly improves diagnostic accuracy of treated VAAs, even in the presence of vascular stents, prostheses, or embolic agents [9]. Additionally, the use of VNC reconstructions, which can effectively replace the True Non-Contrast phase, may contribute to overall dose reduction [4,8]. Despite the growing availability of spectral CT technologies, their application in VAAs has not been comprehensively addressed in a dedicated, clinically oriented review.

Our study aims to provide an overview of current and emerging applications of DECTA in the management of VAAs, integrating technical principles with practical imaging strategies to address specific diagnostic and follow-up challenges.

This narrative review was based on a non-systematic literature search of PubMed and Embase, focusing on English-language articles published up to June 2025.

The search utilized keywords such as “Dual-Energy CT”, “Spectral CT”, and “Photon-Counting CT” in combination with “Visceral Artery Aneurysms” and included peer-reviewed original research, systematic reviews, and meta-analyses addressing dual-energy CT, spectral CT, and Photon-Counting CT in vascular and aneurysmal imaging; case reports without technical data and non-English articles were excluded.

## 2. Visceral Artery Aneurysms: Diagnostic and Therapeutic Insights

VAAs are a rare but clinically significant condition, with an estimated incidence ranging from 0.01% to 0.2% [3,10,11,12].

Splenic artery aneurysms represent the most common type of VAAs, accounting for up to 60% of cases, followed by hepatic, celiac, superior mesenteric, and renal arteries [1,2,11,13].

Approximately 22–25% of VAAs present as acute emergencies due to rupture, a condition associated with high mortality rates, ranging from 25% to 100%. The risk of rupture is particularly elevated in cases of pseudoaneurysms or during pregnancy [14,15,16]. Diagnosis is based on several imaging modalities [1,11,17,18]. CTA is currently considered the gold standard modality for the evaluation of VAAs and for treatment planning, providing detailed assessment of location, morphology (saccular or fusiform), maximum diameter, presence of intraluminal thrombus or wall calcifications, angulation of the vessel origin, presence and extent of vascular collateralization and signs of end-organ ischemia [11,19,20]. Treatment management of VAAs must be tailored according to anatomical and morphological characteristics, clinical stability, and institutional resources [11].

Over recent years, international and national vascular societies have established evidence-based recommendations to guide treatment decisions [21]. In particular, the Society for Vascular Surgery (SVS), the European Society for Vascular Surgery (ESVS), the joint Italian guidelines of the Society for Vascular and Endovascular Surgery and the Society of Medical and Interventional Radiology (SICVE/SIRM), and the Cardiovascular and Interventional Radiological Society of Europe (CIRSE) outline specific indications [22,23,24]. Treatment is generally recommended in symptomatic cases, in presence of rupture, and in asymptomatic lesions exceeding specific diameter thresholds depending on its anatomical location [22,23,24]. Additional indications include aneurysms in women of childbearing age, Visceral Artery PseudoAneurysms (VAPAs), and high-risk lesions [22,23,24,25].

Endovascular approach is considered the first-line treatment for most elective and emergency cases [15,16]. Endovascular strategies include coil embolization performed with various techniques, including “sac-packing” and “sandwich” embolization, deployment of covered stents to preserve arterial flow, flow-diverting stents, and vascular plugs for complex anatomies [11,26]. Open surgery remains indicated in cases of unfavorable vascular anatomy, massive rupture, or hemodynamic instability [4,5]. According to recent reviews, the endovascular approach offers the advantages of reduced hospitalization, lower mortality, and a decreased incidence of major complications; nevertheless, it requires careful and continuous imaging follow-up [15].

## 3. Spectral CT Acquisition Techniques

DECT enables the acquisition and reconstruction of spectral datasets derived from two distinct X-ray energy spectra. In addition to conventional kiloVolt peak (kVp) images, which are comparable to those obtained with Single-Energy Computed Tomography (SECT), DECT provides spectral datasets allowing for material decomposition, virtual monoenergetic imaging, and improved tissue characterization. These capabilities enhance lesion conspicuity, reduce beam-hardening artifacts, enable iodine mapping and quantitative assessment of contrast uptake, and support more accurate differentiation of materials such as calcium, iodine, uric acid, and fat [27,28,29]. Overall, DECT offers significant diagnostic advantages while maintaining workflow integration within standard CT protocols.

Different technological approaches have been developed to obtain dual-energy information, including dual-layer detectors, rapid kVp-switching systems, dual-source configurations, and split-filter techniques [4,27]. A summary of DECT acquisition techniques is provided in Table 1.

### Photon-Counting CT

The most recent and natural evolution of DECT technology is Photon-Counting Computed Tomography (PCCT), which employs Photon-Counting Detectors (PCDs) to record each individual incident photon and measure its energy [30,31,32]. This approach results in superior image quality and spatial resolution, making DECT and PCCT complementary technologies with promising potential for future innovations in advanced imaging [33,34]. PCCT facilitates more accurate material decomposition, providing virtually monochromatic images and improved beam-hardening artifact correction [9,10]. PCCT has also the potential to simultaneously differentiate multiple contrast agents, including K-edge imaging for materials such as gold or gadolinium [30]. Other clinical advantages include radiation dose reduction while enhancing image quality and intrinsic multienergy imaging with direct material quantification without the need for pre-contrast scans [31]. Initial clinical applications of PCCT have been explored in cardiovascular imaging, thoracic, musculoskeletal, and oncologic fields [33,34,35,36,37,38]. While PCCT is entering early clinical adoption, its application to VAAs is still not part of routine and should currently be regarded as a promising future technology, with advantages in spatial resolution and material characterization.

## 4. Spectral CT Postprocessing Techniques in Vascular Pathology

### 4.1. Virtual Non-Contrast (VNC)

DECT enables the creation of VNC images by identifying and subtracting iodine from contrast-enhanced acquisitions [9,39]. These images are designed to closely mimic True Non-Contrast (TNC) scans. Numerous studies have demonstrated that VNC images can reliably substitute for TNC images, potentially eliminating the need for a dedicated non-contrast phase in multiphasic protocols [8,9]. This substitution brings significant advantages, especially in vascular imaging, where minimizing both radiation exposure is crucial, particularly for patients requiring repeated follow-up scans [40,41,42,43]. In VNC imaging, each voxel is decomposed into its primary constituent materials—typically soft tissue, iodine, and fat—enabling the reconstruction of both VNC and iodine distribution maps. VNC demonstrates high diagnostic reliability in detecting EVAR endoleaks [44,45,46] In these settings, VNC imaging has shown diagnostic accuracy comparable to TNC imaging in up to 95% of cases [45], while enabling an up to 61% reduction in radiation dose [46]. The accuracy of VNC images is limited by the timing of contrast acquisition, with significant variability in attenuation values when VNC images are reconstructed from arterial versus delayed phases [47,48].

### 4.2. Iodine Maps

Iodine maps are post-processing tools based on the principle of material decomposition that enable the visualization and quantification of iodine distribution within different tissues both in qualitative (color overlay) and quantitative terms (mgI/mL) [27,49]. The resulting iodine map provides a color-coded or grayscale image, reflecting iodine concentration and perfusion within vascular structures and lesions [50]. Such reconstructions have been found particularly useful in vascular pathologies, such as for endoleak [51,52] or pulmonary embolism detection [53]. Iodine maps also offer significant reductions in contrast and radiation dose and provide robust artifact suppression, supporting earlier and more confident diagnosis even in challenging or artifact-prone scenarios and optimizing clinical workflows, with quantitative performance beneficial for both diagnostics and follow-up [49,50,54].

### 4.3. Virtual Non-Calcium

Virtual Non-Calcium (VNCa) algorithms enable selective subtraction of calcium without affecting iodine-based intravascular contrast or adjacent soft tissues, thereby enhancing vascular visualization [55,56]. These algorithms are particularly useful in the assessment of vessel stenosis caused by calcified plaques, where dense calcifications often induce blooming, streak, and beam-hardening artifacts that obscure vascular lumen, potentially resulting in unnecessary treatments [56,57,58]. VNCa-based techniques have already proven to be effective in improving diagnostic accuracy of carotid artery stenosis assessment, providing a reliable non-invasive alternative to DSA reference standards [57,58,59].

### 4.4. Virtual Monoenergetic Images (VMIs)

Virtual Monoenergetic Images (VMIs) are spectral CT reconstructions that simulate the attenuation characteristics of monochromatic X-ray beams [60,61]. Energy levels typically range between 40 and 200 keV, allowing a wide range of contrast and artifact management options in vascular imaging. Low-energy VMIs (typically 40–70 keV) enhance iodine contrast due to their proximity to the K-edge of iodine (33.2 keV), improving the conspicuity of vascular structures and lesions with subtle enhancement [62,63]. However, this often comes at the cost of higher image noise and a lower Contrast-to-Noise Ratio (CNR) [60]. High-energy VMIs (typically 100–150 keV) reduce image noise and minimize artifacts from hyperattenuating structures such as metallic stents, calcified plaques, embolization coils, and surgical clips [60,61,62,63,64].

These reconstructions are particularly useful in several vascular applications:

- Low-contrast volume protocols, especially in patients with renal impairment or when using slow injection rates [64].
- Detection and delineation of poorly enhancing lesions, such as hypovascular tumors or inflamed vessel walls [62].
- Endoleak detection after endovascular aneurysm repair, where subtle iodine leaks may be not detected in conventional images [46,52,64].
- Stent evaluation, where improved luminal contrast enhances the assessment of luminal patency and in-stent abnormalities [9].
- Coronary artery imaging and myocardial perfusion, where enhanced contrast resolution improves detection of coronary lesions and perfusion abnormalities [63,65,66].

Phantom studies have demonstrated that high-keV VMIs improve the accuracy of in-stent luminal diameter measurements and increase diagnostic confidence in detecting in-stent restenosis [65,67,68].

### 4.5. Material Decomposition

Material Decomposition (MD) is a core functionality of DECT, allowing differentiation and quantification of specific materials based on their unique X-ray attenuation properties [4,9]. Depending on the hardware configuration, DECT scanners use either two-material or three-material decomposition algorithms. Two-material decomposition, typical of single-source systems, models voxels as mixtures of two base substances (often water and iodine), enabling iodine quantification and VNC reconstructions. Three-material decomposition allows separation of three substances: water, calcium, and iodine in angiographic studies, or fat, iodine, and soft tissue in abdominal CT [4,5,50]. Diagnostic performance remains high even in heavily calcified vessels, especially in larger vessels and high-iodine areas, though the technique is more limited in smaller vessels [56,59,69,70,71]. An overview of the main DECT post-processing tools and their specific applications in vascular imaging is provided in Table 2.

## 5. DECTA in the Detection of Visceral Aneurysms

The clinical applications of DECTA in VAAs can be categorized according to their level of validation. Improved aneurysm detection, lesion conspicuity, and material differentiation using VMIs and material decomposition are well-validated applications. Other applications, including iodine quantification for wall characterization and rupture risk assessment, should be considered emerging, as they are largely extrapolated from aortic aneurysm literature and supported mainly by preliminary or retrospective data.

### 5.1. VAAs: Diagnostic Challenges

VAAs may be true aneurysms, encompassing all three arterial wall layers, or pseudoaneurysms, representing a contained, extra-luminal hematoma resulting from wall disruption that communicates with the arterial lumen [11,72]. True aneurysms often reflect underlying atherosclerosis, post-inflammatory changes, or fibromuscular dysplasia, whereas pseudoaneurysms are most frequently iatrogenic, post-trauma, or post-inflammatory in origin [3,11,72,73].

Their often silent clinical course belies the considerable risk of rupture. Thus, early accurate identification is critical for mitigating the significant morbidity and mortality associated with missed or delayed diagnosis [3,11]. Conventional CT has long been the mainstay of imaging, but DECT now enables superior material characterization, artifact reduction, and quantitative tissue assessment [74]. Relevant imaging features extend beyond size and location to include neck morphology (narrow versus wide, as this influences treatment selection), parenchymal effects (e.g., biliary compression in hepatic artery aneurysms and pancreatic compression in hilar splenic aneurysms), and the relationship to adjacent organs. These aspects are critical for avoiding intra- or post-procedural complications such as bowel ischemia, post-embolization pancreatitis, or hepatic insufficiency [3,11,72]. Differential diagnosis commonly includes hyperattenuating parenchymal lesions, contrast-enhancing masses, and vascular variants. Recent series highlight that diagnosis is frequently delayed or missed [72]. A study reported up to 58% of visceral pseudoaneurysms were not detected on initial CT due to suboptimal imaging protocols masking artifacts or lack of radiologist awareness and training [75]. Additional complexity is introduced by artifacts from surgical materials or intricate adjacent anatomy [76]. In this context, DECTA provides additional diagnostic confidence by improving lesion conspicuity and material characterization beyond size-based assessment.

Given the rarity of VAAs, the evidence discussed in this section includes a heterogeneous body of literature, ranging from validated clinical studies to small retrospective series. These data are presented with the intention of illustrating emerging applications rather than establishing definitive clinical recommendations.

### 5.2. Enhanced Aneurysm Detection with VMIs

**Figure 1:**
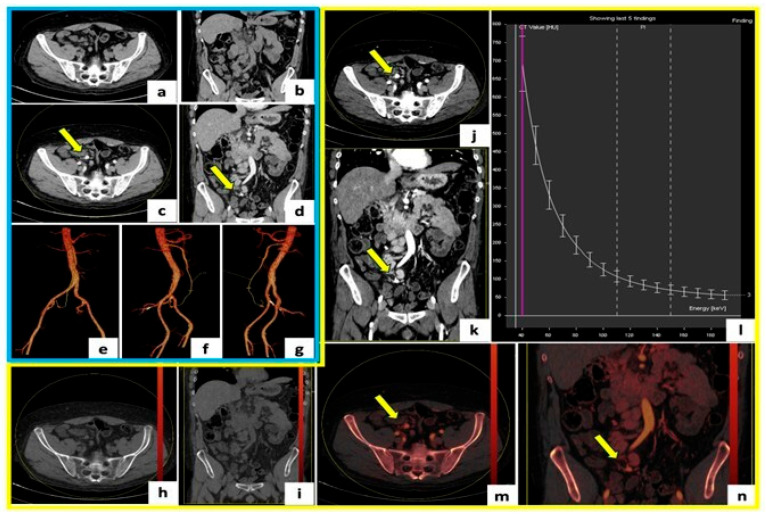
DECTA of a 47-year-old patient with an incidental 9 mm ileocolic artery aneurysm. (**a**–**d**) Axial and coronal unenhanced and arterial-phase images. (**e**–**g**) Volume rendering (VR) reconstructions for arterial branch identification and endovascular planning. (**h**,**i**) Virtual Non-Contrast (VNC) images derived from arterial-phase. (**j**,**k**) Monoenergetic Plus images at 40 keV to enhance contrast and detect small aneurysms. (**l**) Monoenergetic Plus map showing Hounsfield Unit (HU) variation with energy levels. (**m**,**n**) Iodine map images highlighting aneurysm iodine uptake.

DECT enables either simultaneous or rapid sequential image acquisition at two distinct X-ray energy levels, allowing for robust material decomposition and the generation of multiple specialized datasets, such as VMIs (Figure 1). VMIs at low energies (typically 40–55 keV) substantially amplify the photoelectric effect for iodine, leading to a marked enhancement of vascular and hypervascular structures. This translates into improved visibility of small, subtle, or partially thrombosed VAAs and VAPAs, particularly those with slow or stagnant flow or when traditional arterial phase timing is suboptimal [77].

Reducing tube voltage increases iodine contrast by 25–70%, permitting significant reductions in contrast medium volume (by up to 40%) while improving contrast between vessels and surrounding tissues [78,79]. These low-energy acquisitions do introduce higher image noise, but this may be effectively mitigated through advanced postprocessing approaches [78,79].

Innovations including hybrid noise reduction and frequency-based blending techniques have further enhanced the clinical reliability of low-keV VMIs, supporting their routine use in complex vascular lesions such as small aneurysms and mural defects [77,79,80].

### 5.3. Plaque vs. Aneurysm: Differentiation with Material Decomposition

A major technical advantage of DECTA is its ability to differentiate the various materials within the aneurysm sac, providing precise distinction between calcified plaque, mural thrombus, and other tissue components [8,39,46]. Through advanced material decomposition algorithms, such as Hard Plaque Imaging (HPI), DECT produces color-coded overlays, typically labelling calcium in red and iodine or contrast medium in blue. When specifically applied to the evaluation of aortic and visceral aneurysms, this technology enables highly accurate identification of mural calcifications embedded within thrombus or the vessel wall [46]. These materials are often difficult to differentiate with SECT, where hyperdense foci may be misinterpreted as blood products, contrast medium, or device-related artifacts [46]. HPI have demonstrated excellent sensitivity and specificity for the detection of calcifications within the aneurysm sac, even surpassing that of conventional grayscale delayed imaging in certain scenarios. This enhances reliability in differentiating chronic mural changes from acute or residual flow components [46].

Clinically, the use of this post-processing algorithm may impact risk stratification, as characterization and quantification of mural calcifications may help in predicting aneurysm wall stability. Furthermore, the semi-automatic subtraction of calcific plaques using HPI facilitates preprocedural evaluation of the true vessel lumen, especially in anatomically complex or heavily calcified aneurysms, where precise measurements and optimal graft apposition are mandatory [50,81]. However, these potential implications for risk stratification are primarily referred to aortic aneurysm studies, and their direct applicability to VAAs has not yet been specifically validated.

### 5.4. Iodine Quantification May Predict Wall Weakness and Rupture

Quantitative iodine uptake within aneurysm walls may indicate neovascularity and wall weakness, which have been identified as potential predictors of rupture primarily in aortic aneurysm studies [50]. Preliminary findings, largely extrapolated from aortic literature, suggest that quantitative iodine uptake within aneurysm walls might serve as a surrogate marker for neovascularity and wall inflammation. However, its specific prognostic value in VAAs remains investigational and requires further validation [50,82]. Microvascular proliferation and inflammation, both of which are associated with impending wall rupture, may be detected by measuring iodine density within the aneurysm wall or adjacent parenchyma on arterial-phase DECTA [50,82,83]. Early studies have indicated threshold values for risk stratification, and ongoing research aims to validate these findings for prognostic use in clinical workflows. Iodine quantification may be further refined by using effective atomic number mapping (Z-eff) images, providing an additional dimension for tissue characterization.

### 5.5. Emergency Detection and Characterization of Ruptured VAAs

Ruptured VAAs are life-threatening emergencies with high mortality rates (25–70% upon rupture), often presenting with hemorrhagic shock or acute abdominal symptoms [72,84].

CTA is the acknowledged reference standard for emergency imaging, allowing rapid assessment of VAAs, associated hemorrhage, vascular anatomy, and planning for intervention [85,86]. Recent DECT advances may improve the diagnostic accuracy and clinical workflow of ruptured VAA. Material-specific postprocessing techniques enhance the detection of active hemorrhage, pseudoaneurysm, or endoleak in a single arterial-phase acquisition, thus reducing scan time and radiation exposure by up to 50–60% [52,78]. However, arterial-phase acquisition remains mandatory in emergency settings, and DECTA should be regarded as an adjunct rather than a replacement for standard multiphasic CTA protocols. Iodine maps are more sensitive than standard images in localizing active bleeding, and VNC images help to distinguish fresh hemorrhage from pre-existing hematoma. Additionally, low-keV VMIs significantly increase the conspicuity of vascular lesions, particularly for small or intermittently bleeding VAPAs [50,69]. However, diagnostic pitfalls remain. The major cause of missed or delayed VAA diagnosis is failure to perform an adequate arterial-phase acquisition, followed by technical deficiencies in postprocessing (such as excessive noise in low-keV images or insufficient utilization of reconstructions) [75]. Operator experience and training are essential, as subtle findings can be overlooked and artifacts may mask VAAs [72]. Current recommendations should emphasize the integration of DECTA into systematic emergency protocols for suspected ruptured VAAs, with mandatory arterial-phase imaging and reconstructions for comprehensive assessment.

## 6. DECTA in Post-Treatment Follow-Up of Visceral Aneurysms

In post-treatment surveillance of VAAs, well-validated applications of DECTA include endoleak detection, device integrity assessment, and artifact reduction. Quantitative iodine analysis and volumetric sac remodeling have currently shown promising results in EVAR and AAA follow-up and should be regarded as investigational when applied to VAAs.

### 6.1. Overview of VAAs Endovascular Treatment Techniques and Follow-Up

#### 6.1.1. Coil Embolization

Metallic coils, used alone or in combination with other embolic agents/device, determine mechanical obstruction and secondary thrombosis either by their thrombogenic fibers and induced inflammatory reaction [11,87,88,89]. The preferred technique depends mostly on aneurysm shape: fusiform VAA is usually treated with the “isolation technique”, which requires the efferent vessel, aneurysm sac, and afferent vessel to be sacrificed [11,87]. Otherwise, saccular VAAs with a narrow neck are treated with the “sac packing technique” [90].

In saccular aneurysm with wide neck, coils can be released after the implant of an uncovered stent in the parent artery to prevent their migration [91,92,93]. In selected cases with thin target arteries or complex anatomy, coils may be placed before stent deployment [93]. Balloon-assisted coil embolization is another technique for treating wide-neck saccular aneurysms [94,95].

#### 6.1.2. Covered and Flow-Diverting Stents

Covered stent placement offers an alternative in selected cases, particularly when preservation of arterial patency is critical and the aneurysm involves proximal or mid-segments of the vessel [11]. However, its use is limited by vessel tortuosity and small caliber, with technical feasibility reported in only 30% of cases [87]. Self-expandable covered stents are usually preferred in tortuous anatomies, like in visceral arteries, to balloon-expandable because they are more flexible and suitable [96,97]. Another useful device is the flow-diverting stent, originally designed for intracranial aneurysm, which has shown promising results in VAAs with multiple side branches [98]. Their low profile, high porosity, and high flexibility promote gradual aneurysm thrombosis while maintaining branch patency [98,99].

#### 6.1.3. Percutaneous Approach

When a saccular aneurysm with narrow neck is located within solid organs, without bowel interposition, an alternative approach to a failed or non-feasible endovascular treatment is the percutaneous approach, with direct needle puncture injecting thrombin, glue, or other liquid embolic agents under real-time imaging control [100,101]. The risk of a percutaneous approach without angiographic control is an extra-aneurysm accidental spread of thrombogenic material in non-target districts with ischemic complications [100].

### 6.2. Surveillance Protocols and DECTA Optimization

Long-term imaging surveillance is a critical component of VAA management after endovascular treatment, requiring accurate detection of complications while minimizing cumulative radiation and contrast exposure.

Key goals of imaging follow-up include the early detection of endoleaks, aneurysm sac enlargement, device migration, or potential issues involving adjacent structures [81].

DECTA is emerging as a valuable imaging modality not only in the initial diagnostic phase but also for long-term surveillance after endovascular repair [8,39,81]. VMI reconstructions, especially in the 70–80 keV range, offer an optimal balance between reducing artifacts from embolization materials and improving visualization of the surrounding vessels [102,103]. While the ideal post-repair imaging protocol remains debated, biphasic or triphasic approaches—typically including unenhanced, arterial, and delayed phases—are commonly used to detect and characterize endoleaks [8,103,104].

Recent evidence suggests that DECTA can achieve diagnostic accuracy comparable to triphasic SECT while enabling substantial radiation dose reductions, with reported savings of around 29% in biphasic protocols and up to 62% in monophasic delayed protocols [105]. Considering the lifelong imaging surveillance required for these patients, such dose reductions are of significant clinical importance [46]. Delayed-phase imaging remains essential for detecting slow-flow type II endoleak, which may be missed on arterial-phase scans [106]. Nevertheless, arterial-phase imaging can play a supplementary role within biphasic DECTA protocols for detecting high-flow type I/III endoleaks and identifying peri-procedural complications such as arterial injury, pseudoaneurysms, or access site issues [46,106].

### 6.3. DECTA for Endoleak Detection

Endoleak typically present as low-attenuation contrast pooling within or102- adjacent to the treated aneurysmal sac [39,46]. DECTA with colored iodine overlay offers additional diagnostic confidence in endoleak detection. This post-processing tool is highly sensitiveness for detecting even small volumes of contrast medium, being equivalent or in some cases surpassing SECT in endovascular treatment surveillance [46,52,64]. Studies have demonstrated higher sensitivity with DECTA (95% vs. 80%) in abdominal aortic aneurysm (AAA) follow-up; however, evidence in VAAs remains limited and is largely extrapolated from aortic populations [64,102,104,105].

Long-standing evidence highlights the advantages of VNC and low-energy VMIs in enhancing endoleak detection while minimizing radiation exposure. Chandarana et al. demonstrated that delayed venous-phase DECTA using VNC and 80 kVp imaging alone identified all endoleaks, achieving a dose reduction from 27.8 to 11.1 mSv, enabling omission of both unenhanced and arterial phases [107]. Similarly, Stolzmann et al. reported that a single delayed DECTA acquisition offered diagnostic accuracy comparable to triphasic SECT, with sensitivity and specificity ranging from 96% to 100% [102].

A recent meta-analysis by Wen et al. confirmed DECTA’s excellent diagnostic accuracy in detecting endoleaks, showing no significant difference between biphasic and monophasic delayed DECTA protocols compared to standard SECT [105].

Low-keV VMIs have emerged as a key factor in DECTA’s diagnostic superiority. Maturen et al. demonstrated higher sensitivity at 55 keV compared to 75 keV [108], and Martin et al. showed improved endoleak detection using VMIs over blended images [64]. Similarly, Kazimierczak et al. found that 40 keV VMIs improved detection by almost 30% over blended images and increased CNRs [104]. Martin et al. and Zeng et al. recommended 40 keV as the optimal energy for reconstruction in suspected endoleak cases [62,64].

Hard Plaque Imaging with DECTA further enhances diagnostic accuracy by distinguishing endoleaks from calcifications [46]. Additionally, combining 40 keV VMIs with deep learning image reconstruction significantly improves visualization of endoleaks over 70 keV VMIs [109].

### 6.4. Characterization and Typing of Endoleaks

Iodine maps and VNC images can differentiate between high-attenuation hematoma from actively extravasating iodine. This capability, first described in the brain, can be similarly applied to body imaging in EVAR but also in follow up of treated VAAs [70]. CT protocol for follow up of treated VAAs has been widely investigated in literature [110]. The usefulness of the arterial phase, however, is controversial. Some authors highlight the importance of the arterial phase, demonstrating higher sensitivity of multiphasic examination protocols for the detection of type I and type III endoleaks, potentially life-threatening and requiring treatment [104,105]. Furthermore, the arterial phase allows the evaluation of abdominal organs’ perfusion and potentially the implementation of appropriate treatment [8]. Other series have shown that, despite its lower sensitivity for low-flow endoleaks, all endoleaks detected in the arterial phase were also visible in the delayed phase, making arterial phase avoidable [52,111].

Compared to SECT, DECTA has a greater ability to differentiate between endoleak types using quantitative spectral parameters. In a study by Charalambous et al., the normalized effective atomic number improvised endoleak index derived from dual-energy datasets demonstrated excellent diagnostic performance, achieving an Area Under Curve (AUC) of 0.867 for predicting aggressive type II endoleaks, with 100% specificity and 60% sensitivity in distinguishing high- from low-risk lesions [112]. These findings may be particularly relevant for VAAs, where low-flow endoleaks are frequently occult on SECT. Nevertheless, these observations are derived from EVAR cohorts and their clinical utility in VAAs should be considered exploratory pending dedicated validation. Moreover, VMIs at 40–55 keV significantly improved endoleak conspicuity compared to standard blended images, as shown by Martin et al. and Kazimierczak et al., with up to a 30% increase in endoleak detection rate and superior Contrast-to-Noise Ratios [64,104].

### 6.5. Device Integrity and Artifact Reduction

**Figure 2:**
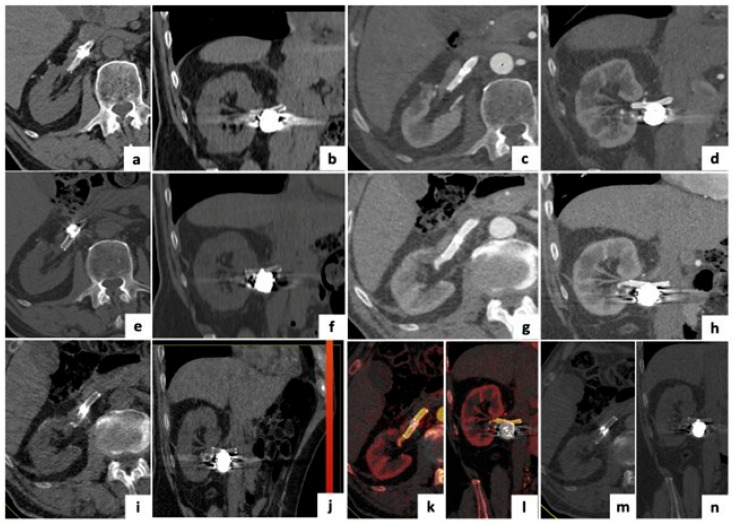
Follow-up imaging of a 78-year-old patient treated with stent-assisted coil embolization of a right renal artery aneurysm. (**a**–**d**) Axial and coronal Single-Energy Computed Tomography (SECT) images 2 months post-treatment, showing artifacts from stent and coils. (**e**–**h**) Axial and coronal Dual-Energy Computed Tomography (DECT) images 1-year post-treatment, with reduced artifacts. (**i**,**j**) DECT post-processed with Virtual Non-Contrast (VNC) application, simulating unenhanced images. (**k**,**l**) Iodine Map application highlighting regular iodine distribution with no aneurysmal leak. (**m**,**n**) Monoenergetic Plus reconstructions at 150 keV, minimizing artifacts while visualizing stent and coil position.

High VMIs, especially at ≥140 keV, significantly mitigate beam-hardening and photon-starvation artifacts caused by coils, stents, and embolic materials, thereby improving the assessment of device apposition, structural integrity, and potential migration [50,62,68]. The artifact suppression is especially relevant in densely packed or large coil volumes, where standard Metal Artifact Reduction (MAR) algorithms often prove insufficient [68]. Combining DECT with MAR techniques, such as iterative reconstruction or VMI+, further enhances image quality, although vessel visualization adjacent to dense coils may remain limited [67] (Figure 2).

Several studies reported the superiority of DECT in evaluating vascular stents, including coronary, peripheral, and mesenteric locations [41,42,47]. Noise-optimized VMIs at 70–80 keV improve stent lumen visualization and reduce blooming artifacts [65,113]. In phantom models, third-generation DECT systems provided optimal imaging for stent diameters <3 mm, while 130–150 keV reconstructions offered improved in-stent lumen clarity with reduced radiation dose [65,114]. Within the broader context of splanchnic vessels, DECT also facilitates the evaluation of transjugular intrahepatic portosystemic shunts, superior mesenteric artery stents, and stents employed during EVAR or Branch EndoVascular repair (BEVAR) procedures, with iodine maps enabling clear differentiation between a patent lumen and thrombus [8,81,115]. Boos et al. reported that MAR alone, while improving stent visualization, often degraded image quality and obscured endoleaks in 60% of patients. In contrast, DECTA with high-keV VMIs reduced near-field artifact variability while preserving endoleak detection [67,116]. As suggested by Winklhofer et al., combining high-keV VMIs with MAR may significantly improve post-procedural imaging of VAAs, allowing precise assessment of treated segments and detection of device migration or in-stent stenoses [68].

### 6.6. Sac Volume Quantification and Remodeling

In post-treatment surveillance of VAAs, DECTA may provide a semi-automated volumetric sac quantification. DECT-based software (DECT is double energy CT) enables accurate segmentation of iodine-enhancing lumen and non-enhancing thrombus, offering superior precision compared to SECT measurements. This volumetric approach facilitates longitudinal assessment of sac remodeling and enhances detection of subtle changes that may precede clinical deterioration [117,118].

Evidence from EVAR studies, where these techniques are more extensively validated, demonstrates excellent inter- and intra-observer reproducibility in DECTA volumetric sac analysis and a strong association between volumetric changes and clinically relevant outcomes, including endoleak development and aneurysm progression [117]. In a five-year longitudinal study of 118 EVAR patients, serial volumetric measurements showed significant correlations between sac volume increase, thrombus attenuation, and endoleak type, using linear mixed models to predict aneurysm evolution [118].

In the context of EVAR, a volumetric sac expansion greater than 10% has been associated with the presence of type II endoleaks: while similar hemodynamic principles may apply to the post-treatment remodeling of VAAs, direct evidence within this specific population remains limited, and such thresholds should be applied with caution in clinical practice [8]. Comparative analyses in abdominal aortic aneurysm follow-up have demonstrated that combining volumetric and diameter metrics improves detection of cases warranting re-intervention (up to 86.4%) compared to either method alone [117,118].

Although the literature on VAAs is limited, the underlying physiologic and imaging principles are analogous, and the application of DECTA volumetry in this context is increasingly advocated. When integrated with additional DECTA capabilities, such as VMI and iodine maps, volumetric analysis enables comprehensive evaluation of sac dynamics, endoleak characterization, and treatment response.

### 6.7. Radiation Dose and Contrast Agent Volume Reduction

DECTA protocols could offer significant advantages in reducing both ionizing radiation exposure and iodine contrast volume during diagnostic and follow-up imaging of VAAs. Multiple studies confirm that a monophasic DECTA arterial acquisition, supplemented by VNC reconstruction and colored iodine overlays, can potentially replace traditional triphasic CT protocols in selected clinical scenarios, reducing overall radiation dose by approximately 29% to 62%, although broader validation in VAA-specific follow-up protocols is still required [8,105,119]. Contrast volume can also be substantially reduced, using approximately 30–70% less iodine while preserving diagnostic image quality via low-keV VMIs [120,121,122]. A prospective randomized trial in patients with abdominal aortic aneurysms showed that low-iodine-dose DECTA reconstructed at 40–60 keV obtained +185% vascular attenuation and +25% CNR compared to standard SECT [122]. Similarly, thoracic aorta studies using low-energy DECTA with 50% iodine reduction demonstrated comparable attenuation and subjective image quality to conventional SECT [123]. These optimizations are particularly impactful for patients requiring lifelong surveillance, such as those treated for VAAs, where cumulative radiation and iodinated contrast burden may increase the risk of radiation-induced malignancy and contrast-induced nephropathy [8,78,124,125]. In summary, for visceral aneurysm imaging, DECTA enables up to ~60% reduction in radiation dose and 30–70% reduction in contrast medium use, without sacrificing diagnostic confidence [8,78,124,125]. These efficiencies support its implementation as a safer and equally accurate alternative to conventional SECT protocols in the long-term monitoring of treated visceral aneurysms.

## 7. Limitations

Despite the growing clinical value of DECTA, several significant limitations currently hinder its widespread adoption in the imaging of VAAs. Firstly, DECT system availability remains limited, as not all hospitals or diagnostic centers are equipped with dedicated dual-energy platforms. This technological disparity leads to significant variability in diagnostic potential and access to advanced imaging protocols. Secondly, even in institutions where state-of-the-art DECT technology is available, optimal utilization requires dedicated training and specific experience [124]. Radiologists must be proficient not only in the handling of the hardware, but also with complex post-processing tools such as iodine maps generation or interpretation of VNC images. Insufficient operator familiarity can result in missed findings, suboptimal protocol selection, or incorrect interpretation of subtle vascular lesions—especially relevant in the detection and follow-up of subtle or complex aneurysms. Another limitation consists in the heterogeneous level of evidence supporting the discussed applications of DECTA in VAAs. Finally, a crucial limitation is the absence of standardized protocols for DECTA imaging of VAAs. There is notable heterogeneity in acquisition parameters, post-processing workflows, and interpretative criteria among different institutions. This lack of consensus can result in inconsistencies in image quality, diagnostic accuracy, and reporting style, and may also hinder multicenter data comparability and the formulation of shared clinical guidelines [50,124].

## 8. Conclusions

Overall, DECTA represents a comprehensive and versatile imaging modality, providing advantages over conventional CT in both detection and post-treatment surveillance of VAAs. Its advancements in lesion characterization, endoleak detection, and reductions in radiation and contrast agent exposure can support clinical decision-making and improve outcomes in VAA patients. However, its widespread adoption remains limited by several factors, including limited availability of dual-energy platforms, the lack of training and standardized imaging protocols.

Future research should focus on validating these applications in multicenter clinical studies and integrating spectral imaging into clinical decision-making algorithms, potentially expanding its role from diagnostic support to prognostic and therapeutic guidance.

Looking forward, PCCT and further refinements of spectral imaging, together with AI- and deep learning–assisted image reconstruction and quantitative imaging biomarkers, have the potential to further improve early detection, risk stratification, and treatment planning for VAAs.

This review provides a structured framework to support radiologists in the appropriate and clinically meaningful use of DECTA in this rare but high-risk condition, highlighting the need to address current barriers in order to maximize its diagnostic potential in VAA detection and follow-up.

## Figures and Tables

**Table 1:** Summary of the main Dual-Energy Computed Tomography (DECT) acquisition technologies, including their technical characteristics, operational requirements, workflow considerations, and key advantages and limitations. Technical parameters may vary across scanner generations and vendors; values are provided as representative examples to illustrate general differences among acquisition technologies.

	Dual Source	Rapid kV Switching	Dual Spin- Computed Tomography	Split-Beam Computed Tomography	Dual-Layer
Description	Two orthogonally mounted x-ray source-detector pairs	Rapid kVp switching between two energy levels	Sequential acquisitions at different tube potentials	*Z*-axis beam splitting using spectral filtration	Dual-layer detectors separating low- and high-energy photons
N. Tubes	2	1	1	1	1
N. Detector arrays	2	1	1	1	1 (Layered)
kVp	70, 80, 90, 100/150 Sn *	80/140	80/135	120 Au-Sn **	120, 14
Maximum tube current/ modulation	1300 1200/800/ Yes	570/ No	580/Yes	800/Yes	1000, 750/ Yes
FoV (cm)	35.5	50	50	50	50
*z*-axis (mm)	57.6	40–80	40–160	38.4	40
Pitch	0.3/1.2	0.5–1.5	up to 1.5	0.25–0.45	0.1–1.8
Fastest Rotation Time (sec)	0.25	0.5	0.27	0.28	0.27
Need to selection	Yes	yes	yes	yes	No
Workflow change	Yes	yes	yes	yes	No
Advantages	Faster rotation time; dose-neutral compared with SECT	Up to a 50 cm field of view	Wider coverage per acquisition	Lower cost compared with dual-source CT	DECT always active at 120/140 kV; rotation time can be as fast as 0.28 s
Limitations	Limited Field of view	No automatic exposure control; higher dose; slower speed	Possible misregistration of high-and low kV images; longer acquisition time; limited use for dynamic contrast-enhanced CT	Slower scanning due to only half of the detector (2 cm) being active per photon energy	Higher radiation dose

* Sn = tin filter applied to the high-energy tube to improve spectral separation in dual-energy CT. ** Au-Sn = gold-tin alloy filter applied to the high-energy tube to enhance spectral separation in dual-energy CT.

**Table 2:** Overview of DECT post-processing tools and their role in vascular imaging.

Reconstruction Technique	Advantage	Application
Material Decomposition	Decompose material into their elemental components	Separation of calcium from iodine Plaque characterization
Iodine Maps	Iodine distribution in tissues	Improved endoleak detection and characterization
Virtual Non-Calcium	Robust calcium subtraction method	Assessing narrow artery stenosis, reducing blooming artifacts from calcified plaque Mitigating the problem of overestimation of stenosis on conventional CTA images
Virtual non contrast	Non-contrast scan can be omitted, reduction in number of phases of examination	Reduction in radiation dose Characteristic of incidental findings in abbreviated examination protocols (without True Non-Contrast phase) Iodine-calcium assessment in tissues and vessel (e.g., endoleak)
Virtual Monoenergetic Images	Mimic the attenuation values of an image obtained with single energy at different Kev values	
Low KeV Virtual Monoenergetic Images	Higher sensitivity for iodine improving its contrast	Improved detection and delineation of poorly enhancing lesions Improved endoleak detection and stent visualization Assessment of coronary vasculature and functional evaluation of the myocardium Contrast dose reduction and salvage of suboptimal contrast examination
High KeV Virtual Monoenergetic Images	Reduction in calcium blooming, beam-hardening and metal artifacts	Reduced artifacts from stent grafts and embolic materials Better visualization of stent lumen Improved visualization of calcified vessels

## Data Availability

No new data were created or analyzed in this study.

## Abbreviations

The following abbreviations are used in this manuscript:

BEVAR	Branched Endovascular Aneurysm Repair
CNR	Contrast-to-Noise Ratio
CTA	Computed Tomography Angiography
DECT	Dual-Energy Computed Tomography
DECTA	Dual-Energy Computed Tomography Angiography
DSA	Digital Subtraction Angiography
EVAR	Endovascular Aneurysm Repair
FOV	Field of View
HPI	Hard Plaque Imaging
MAR	Metal Artifact Reduction
MD	Multidetector
PCCT	Photon-Counting Computed Tomography
SECT	Single-Energy Computed Tomography
TNC	True Non-Contrast
US	UltraSonography
VAA	Visceral Artery Aneurysm
VAPA	Visceral Artery Pseudoaneurysm
VMI	Virtual Monochromatic Imaging
VNC	Virtual Non-Contrast
VNCa	Virtual Non-Calcium
